# Genome Sequences of Four Vibrio parahaemolyticus Strains Isolated from the English Channel and the River Thames

**DOI:** 10.1128/MRA.00392-19

**Published:** 2019-06-13

**Authors:** Lucy Witherall, Sariqa Wagley, Clive Butler, Charles R. Tyler, Ben Temperton

**Affiliations:** a School of Biosciences, University of Exeter, Exeter, United Kingdom; University of Maryland School of Medicine

## Abstract

Vibrio parahaemolyticus is the lead causative agent for seafood-borne human gastroenteritis. While its occurrence has traditionally been uncommon in Europe and the United Kingdom, rising sea surface temperatures have resulted in an increased prevalence. Here, we present the complete genome sequences of four novel V. parahaemolyticus strains isolated in the United Kingdom.

## ANNOUNCEMENT

Vibrio parahaemolyticus is a ubiquitous marine bacterium and an important causative agent for human gastroenteritis (1). It is found in seawaters where temperatures exceed 15°C (2); thus, V. parahaemolyticus abundance and high rates of infection have traditionally been associated with outbreaks in Asia, Africa, South America, and the United States (3–5). However, rising sea surface temperatures have resulted in an increased prevalence in Europe and the United Kingdom (6, 7). In the midst of a warming climate, it is important to map the genetic profile of V. parahaemolyticus to better understand how to prevent and treat human infection. Here, we present the genome sequences for four UK environmental V. parahaemolyticus isolates, isolated as follows: (i) EXE V18/004 from Ostrea edulis at Chichester Harbor (2018), (ii) V12/024 from Crassostrea gigas at Weymouth (2012), (iii) V05/313 from Eriocheir sinensis in the River Thames (2005), and (iv) V05/027 from an unknown shellfish source in Southampton (2005). Strains EXE V18/004 and EXE V13/004 were isolated at the University of Exeter, while strains V12/024 and V05/027 were isolated at Cefas Weymouth Laboratories. All four of these strains were isolated directly from shellfish following previously described methods (2, 8). Strain V05/027 was donated to Exeter University from Cefas Weymouth laboratories.

V. parahaemolyticus was initially identified based on colony morphology on selective agar (marine agar and thiosulphate citrate bile sucrose agar) and by PCR targeting the *toxR* region (9). Isolates were grown in marine broth (10) at 37°C overnight, and 1 ml of each culture (∼10^9^ cells · ml^−1^) was added directly to a Qiagen DNeasy PowerWater kit (Germany) for DNA extraction, followed by library preparation for short-read (2 × 250 bp; Illumina, San Diego, CA) and long-read (MinION; Oxford Nanopore Technologies) sequencing. For short-read libraries, DNA was quantified in triplicate with the Quant-iT double-stranded DNA (dsDNA) high-sensitivity (HS) assay in an Ependorff AF2200 plate reader. Genomic DNA libraries were prepared using a Nextera XT library prep kit (Illumina) following the manufacturer’s protocol with the following modifications: 2 ng of DNA instead of 1 ng was used as the input, and PCR elongation time was increased from 30 s to 1 min. DNA quantification and library preparation were carried out on a Hamilton Microlab Star automated liquid handling system. Pooled libraries were quantified using the Kapa Biosystems library quantification kit for Illumina on a Roche LightCycler 96 quantitative PCR (qPCR) machine. Libraries were sequenced on an Illumina HiSeq instrument. Long-read sequencing was performed in-house using a multiplexed SQK-LSK108 library preparation and sequenced on a FLO-MIN106 flow cell, following Oxford Nanopore Technologies protocol (11). Short reads were adapter trimmed using Trimmomatic v3.0 (12) with a sliding window quality cutoff of Q15. Long-read sequences were base called using the Guppy v2.3.5 FlipFlop algorithm and then demultiplexed and adapter trimmed using Porechop (https://github.com/rrwick/Porechop) with the following settings: -require_two_barcodes -discard_unassigned -discard_middle. Hybrid genome assembly was performed using Unicycler v0.4.7 (13), and each assembly was uploaded to the Integrated Microbial Genomes (IMG) platform (14), developed by the Joint Genome Institute (JGI; USA) for annotation. Short-read coverage was calculated using BBMap v38.22 (https://sourceforge.net/projects/bbmap/) with the following settings: idfilter=0.95 covstats=covstats.txt. Long-read coverage was calculated by first using minimap2 v.2.17 (https://github.com/lh3/minimap2) and SAMtools v.1.9 (http://samtools.sourceforge.net/) to create a bam file (minimap2 -t 16 -ax map-ont <assembly> <long_reads> | samtools view -F 4 -buS | samtools sort -o long.sorted.bam) and then using BBMap’s pileup.sh to calculate coverage (pileup.sh in=long.sorted.bam ref=ref.fa out=long.covstats.txt). Default parameters for all software were used unless otherwise noted.

Each strain was found to have two chromosomes (Table 1), one with an average size of 3.3 Mbp and a smaller one with an average size of 1.8 Mbp, which is consistent with the literature (15). Two strains, EXE V18/004 and V05/313, were found to contain plasmids with sizes of 49,878 bp and 71,936 bp, respectively.

**TABLE 1:** Chromosome length, sequence run statistics, and assembly statistics for the Vibrio parahaemolyticus strains described in this study

V. parahaemolyticus strain	No. of contigs	Chromosome length (chromosome 1; chromosome 2 [bp])	No. of MinION reads	Median MinION read length (bp)	Median short-read coverage (chromosome 1; chromosome 2 [×])	Median long-read coverage (chromosome 1; chromosome 2 [×])	G+C content (%)	No. of protein-coding genes
EXE V18/004	5	3,263,543; 1,747,363	241,527	1,943	38; 30	187; 151	45	4,524
V12/024	2	3,315,999; 1,877,872	18,595	17,487	27; 22	61; 58	45	4,604
V05/313	3	3,319,614; 1,940,186	1,116,682	1,173	53; 42	784; 636	45	4,738
V05/027	2	3,438,892; 1,705,150	168,588	3,832	62; 51	135; 111	45	4,669

### Data availability.

Assembled and annotated genomes are publicly available from JGI IMG/M (https://img.jgi.doe.gov/) using the following taxon identifiers (IDs): 2816332655 (V. parahaemolyticus EXE V18/004), 2816332656 (V. parahaemolyticus V12/024), 2816332657 (V. parahaemolyticus V05/313), and 2816332658 (V. parahaemolyticus V05/027). Read data are available from the European Nucleotide Archive under the following strain names and accession numbers: V. parahaemolyticus EXE V18/004, ERS3342146; V. parahaemolyticus V12/024, ERS3342147; V. parahaemolyticus V05/313, ERS3342148; and V. parahaemolyticus V05/027, ERS3342149.

## References

[1] Nelapati S, Nelapati K, Chinnam BK 2012 *Vibrio parahaemolyticus*—an emerging foodborne pathogen. Vet World 5:48–63. doi:10.5455/vetworld.2012.48-63.

[2] Powell A, Baker-Austin C, Wagley S, Bayley A, Hartnell R 2013 Isolation of pandemic *Vibrio parahaemolyticus* from UK water and shellfish produce. Microb Ecol 65:924–927. doi:10.1007/s00248-013-0201-8. 23455432

[3] Kalatzis PG, Castillo D, Katharios P, Middelboe M 2018 Bacteriophage interactions with marine pathogenic vibrios: implications for phage therapy. Antibiotics (Basel) 7:E15. doi:10.3390/antibiotics7010015. 29495270 PMC5872126

[4] Ceccarelli D, Hasan NA, Huq A, Colwell RR 2013 Distribution and dynamics of epidemic and pandemic *Vibrio parahaemolyticus* virulence factors. Front Cell Infect Microbiol 3:97. doi:10.3389/fcimb.2013.00097. 24377090 PMC3858888

[5] Nair GB, Ramamurthy T, Bhattacharya SK, Dutta B, Takeda Y, Sack DA 2007 Global dissemination of *Vibrio parahaemolyticus* serotype O3:K6 and its serovariants. Clin Microbiol Rev 20:39–48. doi:10.1128/CMR.00025-06. 17223622 PMC1797631

[6] Wagley S, Koofhethile K, Wing JB, Rangdale R 2008 Comparison of *V. parahaemolyticus* isolated from seafoods and cases of gastrointestinal disease in the UK. Int J Environ Health Res 18:283–293. doi:10.1080/09603120801911064. 18668416

[7] Martinez-Urtaza J, Bowers JC, Trinanes J, DePaola A 2010 Climate anomalies and the increasing risk of *Vibrio parahaemolyticus* and *Vibrio vulnificus* illnesses. Food Res Int 43:1780–1790. doi:10.1016/j.foodres.2010.04.001.

[8] Wagley S, Koofhethile K, Rangdale R 2009 Prevalence and potential pathogenicity of *Vibrio parahaemolyticus* in Chinese mitten crabs (*Eriocheir sinensis*) harvested from the River Thames Estuary, England. J Food Prot 72:60–66. doi:10.4315/0362-028X-72.1.60. 19205465

[9] Kim YB, Okuda J, Matsumoto C, Takahashi N, Hashimoto S, Nishibuchi M 1999 Identification of *Vibrio parahaemolyticus* strains at the species level by PCR targeted to the *toxR* gene. J Clin Microbiol 37:1173–1177. 10074546 10.1128/jcm.37.4.1173-1177.1999 PMC88669

[10] ZoBell CE 1941 Studies on marine bacteria. I. The cultural requirements of heterotrophic aerobes. J Mar Res 4:42.

[11] Oxford Nanopore Technologies. 2018 1D native barcoding genomic DNA (With EXP-NBD103 and SQK-LSK108). Package insert. Oxford Nanopore Technologies, Oxford, United Kingdom.

[12] Bolger AM, Lohse M, Usadel B 2014 Trimmomatic: a flexible trimmer for Illumina sequence data. Bioinformatics 30:2114–2120. doi:10.1093/bioinformatics/btu170. 24695404 PMC4103590

[13] Wick RR, Judd LM, Gorrie CL, Holt KE 2017 Unicycler: resolving bacterial genome assemblies from short and long sequencing reads. PLoS Comput Biol 13:e1005595. doi:10.1371/journal.pcbi.1005595. 28594827 PMC5481147

[14] Chen I-M, Chu K, Palaniappan K, Pillay M, Ratner A, Huang J, Huntemann M, Varghese N, White JR, Seshadri R, Smirnova T, Kirton E, Jungbluth SP, Woyke T, Eloe-Fadrosh EA, Ivanova NN, Kyrpides NC 2019 IMG/M v.5.0: an integrated data management and comparative analysis system for microbial genomes and microbiomes. Nucleic Acids Res 47:D666–D677. doi:10.1093/nar/gky901. 30289528 PMC6323987

[15] Okada K, Iida T, Kita-Tsukamoto K, Honda T 2005 Vibrios commonly possess two chromosomes. J Bacteriol 187:752–757. doi:10.1128/JB.187.2.752-757.2005. 15629946 PMC543535

